# Diagnostic Value of Galectin-3 in Distinguishing Invasive Encapsulated Carcinoma from Noninvasive Follicular Thyroid Neoplasms with Papillary-Like Nuclear Features (NIFTP)†

**DOI:** 10.3390/cancers13122988

**Published:** 2021-06-15

**Authors:** Guodong Fu, Olena Polyakova, Ronald S. Chazen, Jeremy L. Freeman, Ian J. Witterick

**Affiliations:** 1 Alex and Simona Shnaider Research Laboratory in Molecular Oncology, Lunenfeld-Tanenbaum Research Institute, Sinai Health System, Mount Sinai Hospital, Toronto, ON M5G 1X5, Canada; Ronald.Chazen@sinaihealth.ca; 2 Joseph and Mildred Sonshine Family Centre for Head and Neck Diseases, Sinai Health System, Mount Sinai Hospital, Toronto, ON M5G 1X5, Canada; Jeremy.Freeman@sinaihealth.ca; 3 Department of Otolaryngology-Head and Neck Surgery, Sinai Health System, Mount Sinai Hospital, University of Toronto, Toronto, ON M5G 1X5, Canada

**Keywords:** thyroid cancer, Gal-3 expression, NIFTP, EFVPTC, lymphocytic thyroiditis (LT), immunohistochemical (IHC) analysis

## Abstract

**Simple Summary:**

The reclassification of NIFTP raised the need for rebuilding the clinical, histologic, cytological and molecular parameters, including re-evaluation of the previously examined biomarkers, for assisting in the diagnosis of this subset of indolent noninvasive tumors from invasive encapsulated follicular variant of papillary thyroid carcinoma (EFVPTC). In this retrospective study, Galectin-3 (Gal-3) IHC staining on patient’s thyroid tissues showed a statistically significant higher cytoplasmic Gal-3 expression in invasive EFVPTC than in NIFTP and other benign subgroups. Our findings refined the diagnostic value of Gal-3 expression as an ancillary marker in identifying NIFTP among encapsulated follicular variant nodules.

**Abstract:**

*Background*: non-invasive follicular thyroid neoplasms with papillary-like nuclear features (NIFTP), which is considered as low-risk cancer, should be distinguished from the malignant invasive encapsulated follicular variant of papillary thyroid carcinoma (EFVPTC). Improved discrimination of NIFTPs from invasive EFVPTCs using a molecular biomarker test could provide useful insights into pre- and post-surgical management of the indeterminate thyroid nodule. Galectin-3 (Gal-3), a β-galactosyl-binding molecule in the lectin group, is involved in different biological functions in well differentiated thyroid carcinomas. The aim of this study was to determine whether Gal-3 expression as a diagnostic marker could distinguish indolent NIFTP from invasive EFVPTC on tissue specimens from surgical thyroid nodules. *Methods*: immunohistochemical (IHC) analysis of cytoplasmic and nuclear Gal-3 expression was performed in formalin-fixed paraffin-embedded (FFPE) surgical tissues in four specific diagnostic subgroups- benign nodules, NIFTPs, EFVPTCs and lymphocytic/Hashimoto’s thyroiditis (LTs). *Results*: cytoplasmic Gal-3 expression (mean ± SD) was significantly increased in invasive EFVPTCs (4.80 ± 1.60) compared to NIFTPs (2.75 ± 1.58, *p* < 0.001) and benign neoplasms (2.09 ± 1.19, *p* < 0.001) with no significant difference between NIFTPs and benign lesions (*p* = 0.064). The presence of LT enhanced cytoplasmic Gal-3 expression (3.80 ± 1.32) compared to NIFTPs (*p* = 0.016) and benign nodules (*p* < 0.001). Nuclear Gal-3 expression in invasive EFVPTCs (1.84 ± 1.30) was significantly higher than in NIFTPs (1.00 ± 0.72, *p* = 0.001), but similar to benign nodules (1.44 ± 1.77, *p* = 0.215), thereby obviating its potential clinical application. *Conclusions*: our observations have indicated that increased cytoplasmic Gal-3 expression shows diagnostic potential in distinguishing NIFTP among encapsulated follicular variant nodules thereby serving as a possible ancillary test to H&E histopathological diagnostic criteria when LT interference is absent, to assist in the detection of the invasive EFVPTC among such nodules.

## 1. Introduction

Thyroid cancer has the most rapidly increasing incidence rate among all major cancers, with a triple increase from 4.5 to 14.4 per 100,000 population during 1974–2013 [1]. It was estimated 52,890 new cases in the United States in 2020 and contributed to 0.36% of all cancer deaths [2,3]. Most primary thyroid cancers are follicular cell-derived epithelial tumors, making up four main pathological carcinoma types: papillary thyroid carcinoma (PTC), follicular thyroid carcinoma (FTC), poorly differentiated thyroid carcinoma (PDTC) and anaplastic thyroid carcinoma (ATC). Medullary thyroid carcinomas (MTC) originate from thyroid parafollicular (C) cells. PTC and FTC are differentiated thyroid cancers, whereas ATC is undifferentiated. PTC constitutes up to 90% of all thyroid malignancies [4], followed by FTC (5–10%), ATC (<2%, typically occurring in the elder patients) and MTC (2%) [5]. As the most common type of well-differentiated thyroid cancer [6], PTC comprises numerous histopathologic variants that are well validated clinically and biologically, including classic variant, follicular variant (encapsulated/well demarcated, with tumor capsular invasion), follicular variant (encapsulated/well demarcated, noninvasive), follicular variant (infiltrative), tall cell variant, hobnail variant, cribriform-morular variant, columnar cell variant and diffuse sclerosing variant [7]. Among all variants, the follicular variant of papillary thyroid carcinoma (FVPTC) is one of the most common diagnoses, representing up to 30% of all thyroid carcinoma cases [8]. FVPTC is subdivided into two subtypes: the infiltrating/diffuse variant (infiltrative FVPTC), which has the metastatic tendency of the classic papillary thyroid carcinoma, and the encapsulated variant (EFVPTC), which may present with no capsular or angiolymphatic invasion or metastases [9,10]. The microscopic diagnostic criteria for EFVPTC are mainly encapsulation or clear demarcation, follicular growth patterns, and nuclear features of papillary carcinoma (enlargement, crowding/overlapping, elongation, irregular contours, grooves, pseudoinclusions and chromatin clearing) [11,12,13,14]. Controversies have existed for a long time about whether all EFVPTC cases should be classified as malignancy [15,16,17]. Evidence suggests that a subset of non-invasive EFVPTC, earlier considered as conventional thyroid cancer, has been demonstrated to be a highly indolent tumor showing an overall risk of recurrence (local, regional and distant) of less than 1% [15,16,18,19,20]. In 2016, a new terminology Non-invasive follicular thyroid neoplasm with papillary-like nuclear features (NIFTP) has been proposed to name the subset of non-invasive EFVPTC [11] to address the indolent behavior of this tumor and avoid the term “carcinoma” and potential over-treatment.

NIFTP presents the following characteristics: (1) main morphological features, including encapsulation or clear demarcation, follicular growth pattern, no well-formed papillae, no psammoma bodies, <30% solid/trabecular/insular growth pattern, nuclear score 2–3, no tumor necrosis, no high mitotic activity; (2) lack of invasion, which separates this tumor from invasive EFVPTC; (3) a very low risk of the adverse outcome when the tumor is non-invasive [11,21,22]. Histopathological examination of the entire tumor remains the gold standard for NIFTP diagnosis. Of note, the initial exclusionary papillae less than 1% that was subjective to apply has been updated to a 0% cut-off to ensure indolent outcome [22,23]. NIFTP is still an evolving diagnosis, and more data need to be established to substantiate this new entity [24]. As neoplasm develops specific alterations at the proteomic and/or genomic level, molecular marker-based ancillary tests at histological or cytological level may be a great asset in improving the accuracy of the diagnosis of NIFTP [4,11,25,26]. Recently, immunohistochemical test of Programmed Death-Ligand 1 expression has been shown to distinguish invasive EFVPTC from the indolent NIFTP and benign nodules [25], and recommended as an auxiliary aid to pathologists [27].

Galectin-3 (Gal-3), a member of the beta-galactoside-binding protein family that is predominantly localized in the cytoplasm, may translocate to the perinuclear membrane, nucleus and/or secreted from the cytoplasm [28,29]. This protein is involved in various physiological and pathological processes, such as cell proliferation, apoptosis, inflammation, cell adhesion, cellular transformation, tumor progression and metastasis of cancer cells [28,30]. Overexpression of *galectin-3* cDNA in vitro generates a transformed phenotype [31]; conversely, inhibition of Gal-3 expression has suppressed tumor growth in mice tumor models [32]. Gal-3 is a p53 physiological target mediating p53-induced apoptosis at the molecular level; therefore, the aberrant expression of Gal-3 blocks the apoptotic program, promoting the development of cancer [33]. Gal-3 is overexpressed in a high proportion of carcinomas, especially of the papillary histotype, but weak or absent from normal or benign thyroid tissue [29,30,34], suggesting its potential biological role in the malignant transformation of thyroid cells. Gal-3 immunohistochemistry has been reported to assist the diagnosis of FVPTC [29,35]; however, the role of Gal-3 expression as an ancillary marker in reclassification to NIFTP has not yet been determined.

The aim of the current study was to determine whether Gal-3 subcellular expression in histological surgical tissues could serve as a useful biomarker to distinguish indolent NIFTP from invasive EFVPTC. 

## 2. Materials and Methods

### 2.1. Patient Specimen

This retrospective cohort study was performed at Sinai Health System, Mount Sinai Hospital (MSH), a University of Toronto-affiliated hospital and a prime referral center for patients with thyroid disorders in Toronto, Canada. A total of 1859 patients who previously underwent thyroidectomy in MSH between 2010 and 2015 were identified following the approved guidelines of the Sinai Health System Research Ethics Board (REB #07-0212-E). The pathologist (OP) reviewed the patient clinical charts, surgical pathology reports and H&E stained sections [25]. The final diagnosis was made in accordance with the WHO’s Classification of Tumors of Endocrine Organs [36], CAP protocol [7,37] and Endocrine Pathology Society Working Group [11]. After the histopathological evaluations based on strictly defined inclusion and exclusion criteria [11,23], a total of 165 archived formalin-fixed paraffin-embedded (FFPE) tissue blocks of thyroid tumors (≥1.0 cm) retrieved from the MSH Tumor Bank were classified into four specific diagnostic subgroups: 42 cases of benign nodules, 41 NIFTPs, 45 invasive EFVPTCs and 37 cases of noninvasive encapsulated FVPTC lesions with co-existing significant lymphocytic/Hashimoto’s thyroiditis (LT) [25,37].

### 2.2. Histological Inclusion/Exclusion Criteria

The detailed inclusion/exclusion criteria were described in recent studies [23,25]. Briefly, for the NIFTP subgroup, the inclusion criteria included major features (encapsulation/clear demarcation, follicular growth pattern, nuclear features of PTC (2–3 points of the 3-point nuclear scoring scheme): enlargement, crowding/overlapping, elongation, irregular contours, grooves, pseudoinclusions, chromatin clearing) [37] and minor features (dark colloid, irregularly shaped follicles, intratumoral fibrosis, “sprinkling” sign, follicles cleft from stroma, multinucleated giant cells within follicles) [11]. The exclusion criteria used for NIFTP were the presence of “true” papillae, psammoma bodies, infiltrative border, tumor necrosis, high mitotic activity, cell/morphologic characteristics of other variants of PTC. EFVPTC with different degrees of capsular transgression exhibited features within an encapsulated or well circumscribed nodule, follicular architecture, PTC nuclear features and papillae [37]. Noninvasive FVPTC with lymphocytic/Hashimoto’s thyroiditis (LT) histologically presented with extensive lymphocytic infiltrate with germinal center formation, lymphocytes predominantly and plasma cells, atrophic follicles with abundant Hürthle cells (large, polygonal cells with abundant granular eosinophilic cytoplasm and large hyperchromatic round to oval regular nucleus). Therefore, LT was placed in a separate category to avoid possible false positive interpretation of a co-existing inflammatory environment as a factor in Gal-3 cytoplasmic expression [37,38,39]. 

### 2.3. Immunohistochemical (IHC) Staining

A representative FFPE section from each tissue block (5 μm thickness) was deparaffinized, hydrated with series of graded alcohol and then antigen retrieval at 115 °C for 3 min was performed in Tris-EDTA buffer (10 mM Tris base, 1 mM EDTA, 0.05% Tween 20, pH 9.0) as described previously [40]. Tissues were treated with 3% H_2_O_2_ in Tris-buffered saline for 8 min to block the endogenous peroxidase activity. Then slides were incubated with a background punisher to block non-specific staining (Biocare Medical, LLC, Concord, CA, USA) for 20 min, and were followed by 1 hr incubation with rabbit anti-Gal-3 antibody at a 1:500 dilution (ab53082, Abcam, Cambridge, MA, USA). The rinsed sections were then incubated with biotinylated anti-rabbit secondary antibody for 20 min, subsequently detected using The VECTASTAIN ABC System (Vector Labs, Burlington, ON, Canada) and peroxidase substrate diaminobenzidine (DAB) as the chromogen until staining signal developed. Sections of a classical variant of PTC known with Gal-3 positivity were included in each batch of immunostaining for Gal-3 antibody incubation to serve as an external positive control as well as for an irrelevant isotype specific IgG in place of the primary antibody as a negative control to exclude a nonspecific staining. All slides were counterstained with hematoxylin and viewed under a light microscope.

### 2.4. Evaluation of Immunohistochemistry

IHC sections were scored as positive if epithelial cells showed immunoreactivity in the cytoplasm and/or nucleus using a bright-field microscope (Olympus BX50; Olympus, Center Valley, PA, USA). Sections were scanned at low (×40) and high powers (×200), and Gal-3 stained cells were scored semi-quantitatively by three independent observers (GF, OP, and RSC). Immunostaining scores were determined based on the percentage positivity and staining intensity as described previously [25]. Percentage positive scores were assigned according to the scale: 0 (≤10%), 1 (11–30%), 2 (31–50%), 3 (51–70%) and 4 (>70%). Staining intensity was scored semi-quantitatively as follows: 0 (none), 1 (mild), 2 (moderate) and 3 (intense). A total score for each cytoplasmic or nuclear staining was then obtained (ranging from 0 to 7) by adding the percentage positivity and intensity scores for each section. The IHC scoring was blinded from the histopathology report. Representative photomicrographs showing each IHC score category were included in the supplementary file (Figure S1). The final score was given as an average of scores using following formula: ((percentage score 1 + intensity score 1) + (percentage score 2 + intensity score 2) + (percentage score 3 + intensity score 3))/3. 

### 2.5. Statistical Analysis

Gal-3 IHC score data were analyzed using IBM SPSS Statistics version24 (SPSS, Chicago, IL, USA, http://www.ibm.com/analytics/us/en/technology/spss/ (accessed on 2 March 2020). Descriptive statistic was used to describe the patient population and inferential statistic was used to test the Gal-3 expression differences between the groups (one-way ANOVA). Gal-3 positivity scores served as the predictor variable and invasion served as the primary outcome variable (invasiveness). Diagnostic accuracy was assessed by plotting receiver operator characteristics (ROC) curve. The cut off points for Gal-3 positivity expression and area under the curve (AUC) were calculated. The optimal cutoff value was chosen as the threshold that maximized the AUC. The clinical utility of biomarkers was assessed using AUC, positive predictive value (PPV), negative predictive value (NPV) for invasiveness. The association between cytoplasmic Gal-3 positivity and invasiveness was examined by odds ratio (OR) using cross-tabulation. For the accuracy assessment of positive cytoplasmic Gal-3 expression in distinguishing NIFTP and invasive EFVPTC, we calculated the likelihood ratios (LR): (LR+ = sensitivity/(1-specificity)) and (LR− = ((1-sensitivity)/specificity). The results were considered statistically significant at *p* ≤ 0.05.

## 3. Results

### 3.1. Patient Characteristics and Clinical Histopathological Features

The patient characteristics and clinical histopathological features of the retrospective study cohort are summarized in Table 1. The median patient age at initial diagnosis was 50 years. (range 16–89) for the subgroup of benign nodules, 55 years. (range 25–80) for NIFTPs, 49 years. (range 19–78) for the invasive EFVPTCs and 52 years. (range 23–75) for the LT subgroup. The majority of patients were females (131, 79%) compared to male’s patients (34, 21%). Clinical, radiologic, or pathologic follow-up data were also reviewed. The median duration of follow-up for the benign subgroup was 6.99 years., NIFTPs 4.10 years., invasive EFVPTC 3.26 years. and LT subgroup 4.13 years. No recurrence for malignant cases was found.

According to the clinical histopathological features, all patients were allocated into four study subgroups- 42 benign nodules (25%), 41 NIFTPs (25%), 45 invasive EFVPTCs (27%) and 37 cases (22%) of encapsulated FVPTC lesions with coexisting significant lymphocytic thyroiditis and no capsular or vascular invasion (LT).

### 3.2. Cytoplasmic Gal-3 Expression Is Increased in Invasive EFVPTC

**Figure 1:**
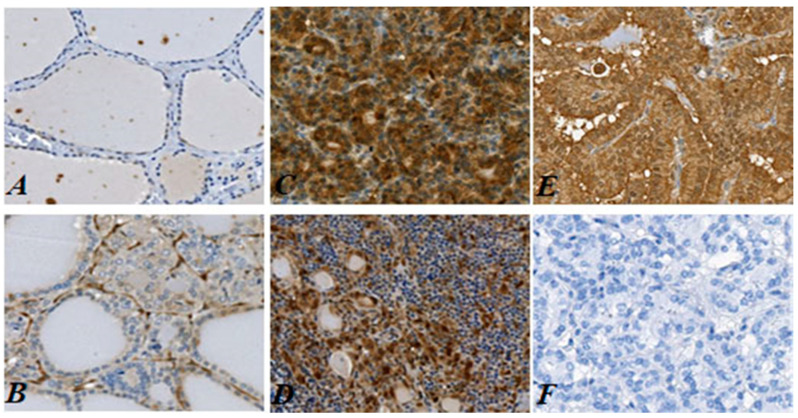
Histological IHC of Gal-3 expression in the tissue sections of Benign Nodules, NIFTP, invasive EFVPTC and LT tissues. (**A**). Lower detectable cytoplasmic Gal-3 expression was observed in the Benign Nodules tissue section. (**B**). Faint cytoplasmic Gal-3 immunostaining was detected in the NIFTP tissue section. (**C**). Moderate to strong cytoplasmic Gal-3 expression was observed in EFVPTC with capsular invasion. (**D**). Mild to moderate cytoplasmic Gal-3 expression was observed in the stained tissue section of LT. (**E**). Positive control, a classic aggressive PTC stained with anti-Gal-3 antibody showed moderate to strong immunostaining. (**F**). Negative control, a classic PTC incubated with isotype specific IgG in place of the anti-Gal-3 antibody did not show detectable immunostaining. All images were shown at original magnification ×200. All images were shown at original magnification ×200.

**Figure 2:**
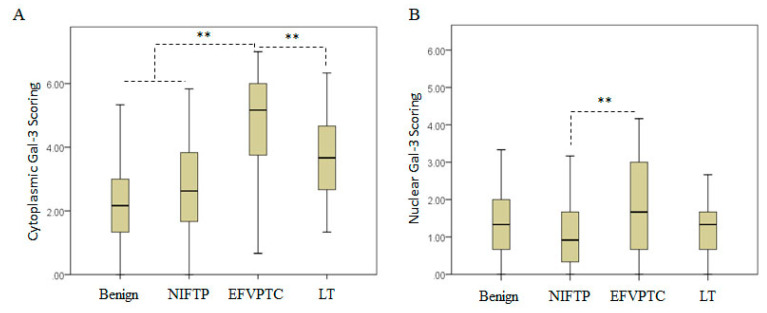
Distribution of Gal-3 expressions in Benign Nodules, NIFTP, EFVPTC and LT tissues. (**A**). Cytoplasmic Gal-3 expression; (**B**). Nuclear Gal-3 expression. The box-and-whisker plot showed the minimum, first quartile, median, third quartile and maximum of each group of data. Double stars (**) denoted a highly significant difference in means between groups at *p* < 0.01 (one-way ANOVA).

Gal-3 IHC analysis was carried out to determine its expression at the subcellular level in thyroid benign nodules, NIFTPs, invasive EFVPTCs and LT subgroup (Figure 1A–D and Figure 2A,B). Gal-3 positive control and the negative control with an isotype specific IgG were stained respectively using thyroid cancer tissue in each batch of immunostaining (Figure 1E,F). Gal-3 expression was mainly observed in tumor cells, whereas the stromal component of tumor did not show positive Gal-3 staining. Cytoplasmic Gal-3 expression (mean ± SD) was significantly increased in invasive EFVPTCs subgroup (4.80 ± 1.60) as compared to NIFTPs (2.75 ± 1.58, *p* < 0.001) and benign nodules (2.09 ± 1.19, *p* < 0.001, Table 2 and Table 3); and there was no significant difference between NIFTPs and benign lesions (*p* = 0.064). The presence of LT also enhanced cytoplasmic Gal-3 expression (3.80 ± 1.32) compared to NIFTPs (*p* = 0.016) and benign nodules (*p* < 0.001). Nuclear Gal-3 expression in EFVPTC (1.84 ± 1.30) was significantly higher than in NIFTPs (1.04 ± 0.72, *p* = 0.001).

However, no significant difference was observed between invasive EFVPTC and benign nodules (1.44 ± 1.07, *p* = 0.215, Table 2 and Table 4), which negated the utility of nuclear Gal-3 expression as a reliable diagnostic aid in detecting invasive EFVPTC.

### 3.3. Cytoplasmic Gal-3 Predictive Value for NIFTP versus Invasive EFVPTC

The accuracy of cytoplasmic Gal-3 expression in distinguishing invasive EFVPTC from NIFTP and benign thyroid nodules was determined by the ROC curve analysis. Cytoplasmic Gal-3 emerged as the strongest predictor of invasive EFVPTC in comparison with nonmalignant tissues (AUC = 0.90, (CI 0.83–0.97), *p* < 0.001) thereby underscoring its potential clinical applicability (Table 5 and Figure S2). With a positive cut off value 3.71 (Table S1), cytoplasmic Gal-3 expression in EFVPTC versus in NIFTP showed a sensitivity of 75.6% (95% CI: 63.9–87.2%), specificity 80.5% (95% CI: 68.6–92.4%), positive predictive value (PPV) 81.0% (95% CI: 69.6–92.3%) and negative predictive value (NPV) 75.0% (95% CI: 62.8–87.2%) (Table 5).

The cross tabulation analysis of diagnostic odds ratio (OR) showed the positive cytoplasmic Gal-3 expression in invasive EFVPTC was 13 times higher risk of having adverse outcome compared to indolent NIFTP (OR = 12.75, 95% CI: 5.88–49.14). The positive likelihood ratio (LR+ = 3.87, 95% CI: 2.21–7.97) indicated the probability in diagnosing as malignant tumors was increased 2.1 times more in specimens with the positive cytoplasmic Gal-3 expression. However, the negative likelihood ratio (LR- = 0.30, 95% CI: 0.14–0.45) showed the probability of malignancy was decreased by 30% in specimens with the low cytoplasmic Gal-3 expression. 

## 4. Discussion

Indeterminate thyroid nodules are highly prevalent in daily clinical practice and represent up to 30% of all clinically assessed thyroid nodules [41], whereas only less than 20% of this group are malignant. Invasive EFVPTC, noninvasive EFVPTC (not meeting criteria for NIFTP diagnosis) and infiltrative FVPTC were designated as malignant lesions, making the diagnosis of thyroid cancer very difficult in cases where histological hallmarks of invasion are not evident [15,16,17,37]. A subset of encapsulated follicular tumors, formerly considered to be noninvasive encapsulated/well demarcated follicular variant of PTC, has been reclassified under a new histological nomenclature, NIFTP [11]. The incidence of NIFTP was as high as 13.3% of all PTC cases in North American and European populations [42] and 16.8% of all well-differentiated thyroid cancers in Ontario, Canada [24]. Histopathological examination of the entire capsulated tumor after its resection according to the rigid diagnostic criteria remains the gold standard for NIFTP diagnosis. Malignant behavior (lymph node and/or distant metastasis) has been reported in NIFTP patients [21,43,44]. NIFTP is not entirely considered as a benign thyroid neoplasm, but correct classification of noninvasive EFVPTC that would qualify NITFP is required to ensure an extremely low rate of adverse oncologic outcomes. Molecular marker testing has risen as an auxiliary tool to distinguish malignant invasive EFVPTCs from more indolent NIFTPs and benign nodules [11,25], potentially assisting pathologists in the management of indeterminate thyroid nodules [27]. Scores on the base of the levels of protein marker expression in thyroid nodules may objectively distinguish malignant lesions from indeterminate thyroid nodules, thereby aiding the correct diagnosis and consequent avoidance of over-treatment of NIFTP lesions when the score is low. Our previous study has shown that the degree of cytoplasmic PD-L1 expression could serve as a useful adjunct to traditional H&E histopathology assessment of such nodules, among which with a low expression of cytoplasmic PD-L1 can be considered as benign nodules or NIFTP [25].

Gal-3 expression has been recognized as a promising diagnostic molecular marker of thyroid malignancy due to its differential expression between thyroid carcinomas and normal or benign thyroid tissues [29,45,46,47]. However, the reclassification of EFVPTC without capsular or vascular invasion to NIFTP not only affects how pathologists evaluate and report this subset of thyroid neoplasms but also raises the need for rebuilding the clinical, histologic, cytologic and molecular parameters for this new entity and accordingly establishing new molecular tests [22]. Therefore, the use of ancillary testing with protein markers previously developed, such as Gal-3 and HBME1, requires to be re-evaluated in the era of NIFTP. In the present study, our data have shown that cytoplasmic Gal-3 expression is significantly increased in invasive EFVPTCs as compared to NIFTPs or benign thyroid nodules in thyroid resection specimens. Concurrently, though there was no significant difference between NIFTPs and benign nodules, cytoplasmic Gal-3 expression in the former was higher than that in the latter, supporting NIFTP cannot be simply considered as a benign lesion. Chronic inflammation can be associated with 30–58% of PTC [48,49]. We also noted that the presence of LT enhances cytoplasmic Gal-3 expression and henceforward the LT increased expression needs to be interpreted with caution. This observation is consistent with the result from another report which showed the increased expression of Gal-3 in an inflammatory environment [50]. Patients with LT were usually under prolonged stimuli from chronic inflammation. The mechanism underlying modulation of Gal-3 expression in thyroid with chronic inflammatory process remains to be determined. Localization of Gal-3 in papillary carcinomas has been reported in both the cytoplasm and nucleus [40,51,52], however, our findings and other’s [29] showed predominant expression of Gal-3 in the cytoplasm in PTC rather than the nucleus. Nuclear expression has been observed in some benign thyroid conditions in our study and reports of others [51,52]. The increased cytoplasmic Gal-3 in invasive EFVPTC might contribute to thyroid cancer development through the induction of the capsular, vascular and/or extrathyroidal invasive activity. The detailed correlation between Gal-3 expression and the degree of invasion was not able to be analyzed since most EFVPTC cases were presented with minimal capsular invasion in the current study. Recently, genetic alterations were intensively studied, such as *BRAF*, *RAS* and *TERT* promoter mutations and *RET/PTC* and *PAX8/PPARγ* rearrangements [53,54]. NIFTPs are commonly detected with the frequent occurrence of RAS mutations and lack of BRAF*^V600E^* and TERT promoter mutations [23]. Whether the level of Gal-3 expression can be associated with such mutational status for better identifying NIFTP requires further investigation. Our observations have suggested that cytoplasmic Gal-3 expression can be considered as an ancillary aid to H&E diagnostic criteria in distinguishing invasive EFVPTC from NIFTP and benign nodules. 

After four decades of steady increase, thyroid cancer incidence rate reached a plateau and possibly started to decline between 2013 and 2020 in the United States [55]. This reverse trend in the incidence of thyroid cancers has been correlating with the increasing understanding of over-diagnosis and the indolent nature of many thyroid nodules that were more likely classified as cancers previously. NIFTP has emerged as a low risk tumor with an indolent clinical course. The present study was focused on evaluating the diagnostic value of the Gal-3 cytoplasmic expression in the histological tissue samples between NIFTP and EFVPTC. To our knowledge, this is the first report showing the diagnostic value of increased cytoplasmic Gal-3 expression in ruling out the indolent NIFTP from invasive EFVPTC. The Gal-3 test proposed here does not replace conventional surgical histopathological examination but represents an auxiliary diagnostic method, especially for cases where morphologic features of invasion are equivocal, that may affect clinical decision-making with regard to completion thyroidectomy, central lymph node dissection, and adjunctive radioiodine therapy. In practice, Gal-3 staining alone add little to histology evaluation when the diagnosis of NIFTP could be achieved via complete resection of the nodules for histological examination of the entire capsule to rule out invasion. However, in pre-surgical fine needle aspiration (FNA) biopsies, NIFTP can belong to any of four categories of the Bethesda System for Reporting Thyroid Cytopathology (TBSRTC), including benign, atypia of undetermined significance or follicular lesion of undetermined significance (AUS/FLUS), follicular neoplasm or suspicious for a follicular neoplasm (FN/SFN) and suspicious for malignancy (SFM) [56]. The definitive diagnosis of NIFTP cannot be made based on the observation of the preoperative cytology specimens, while molecular tests would be highly useful to improve the accuracy in the diagnostics of NIFTP in FNA biopsies. Gal-3 test could have clinically significant utility in assisting in preoperative diagnosis if it were successfully applied to cytology specimens [57]. We are aware of the limitation of our study which is based upon a single patient cohort from a single tertiary care center. Future studies in a larger patient cohort from multiple centers are needed to validate our observations and conclusions. Furthermore, the NIFTP cases were re-classified based on a thorough review of pathology reports and assessment of H&E slides in this study. NIFTP diagnosis is challenging for pathologist and a potential misclassification error might exist particularly when specimens were managed in a way the entire tumor capsule could not be fully assessed for invasion based on pathology review of slides. We are also cognizant that the clinical outcome analysis for each subtype was limited due to incomplete follow-up information, hence the possible association of cytoplasmic Gal-3 expression with the long-term prognoses of NIFTP verses invasive EFVPTC remains further investigation. 

## 5. Conclusions

In conclusion, our data have demonstrated that increased cytoplasmic Gal-3 expression can (i) significantly distinguish indolent NIFTP from invasive EFVPTC; (ii) assist in the early detection of thyroid tumors with aggressiveness and potential metastatic spread which can be suspected by the increased cytoplasmic Gal-3 expression; (iii) support its clinical application as a useful ancillary test to H&E histopathological diagnostic assessment in distinguishing invasive EFVPTC from NIFTP when there is no significant interference from LT. 

## Figures and Tables

**Table 1:** Demographics and clinicopathologic features of the patient cohort.

Characteristics	Benign	Neoplasm		LT *	Total Cases
Pathological Diagnosis	Hyperplasia & Adenoma	NIFTP	Invasive EFVPTC	Lymphocytic Thyroiditis	All Patients
	*n* = 42	*n* = 41	*n* = 45	*n* = 37	*n* = 165
**Age, Mean (range), years**	49.0 (15.8–88.9)	52.1 (22.7–79.5)	48.6 (19.0–77.5)	51.0 (22.8–74.8)	50.1 (15.8–88.9)
**Sex, No. (%)**					
**Female**	34	34	35	28	131
<45 years	13	12	13	9	47 (28)
≥45 years	21	22	22	19	84 (51)
**Male**	8	7	10	9	34
<45 years	1	2	5	2	10 (6)
≥45 years	7	5	5	7	24 (15)
**Tumor size, mean(range), cm**	3.6 ** (1.2–7.4)	2.6 (1.1–5.9)	2.9 (1.0–8.8)	3.0 (1.0–6.4)	3.0 (1.0–8.8)
**Surgery**	42	41	45	37	165
Lobectomy	3	1	1	1	6
Total thyroidectomy	29	40	44	36	149
**Follow-up**					
Mean (years)	6.99	4.10	3.26	4.13	4.65

* Advanced thyroiditis was separated from other noninvasive categories due to co-existing significant lymphocytes/Hashimoto’s thyroiditis. ** Average of tumor size was calculated from 27 cases out of 42 benign patients because the information of the remaining 13 cases was not available.

**Table 2:** Gal-3 expression level in cytoplasm and nucleus in tissues from Benign, NIFTP, EFVPTC and Lymphocytic thyroiditis (LT).

Groups	N	Cytoplasmic Gal-3			Nuclear Gal-3		
		Mean ± SD	95% CI		Mean ± SD	95% CI	
			Lower Bound	Upper Bound		Lower Bound	Upper Bound
Benign	42	2.09 ± 1.19	1.72	2.46	1.44 ± 1.07	1.10	1.77
NIFTP	41	2.75 ± 1.58	2.25	3.25	1.04 ± 0.72	0.79	1.23
EFVPTC	45	4.80 ± 1.60	4.32	5.28	1.84 ± 1.30	1.45	2.23
LT	37	3.80 ± 1.32	3.32	4.21	1.23 ± 0.85	0.95	1.51

**Table 3:** Comparison of cytoplasmic Gal-3 expression among groups of Benign, NIFTP, EFVPTC and Lymphocytic thyroiditis (LT).

(I) Groups	(J) Groups	Mean Difference (I–J)	Std. Error	*p* Value	95% CI	
					Lower Bound	Upper Bound
Benign	NIFTP	−0.659	0.316	0.162	−1.479	0.161
	EFVPTC	−2.737 *	0.312	<0.001	−3.547	−1.927
	LT	−1.703 *	0.328	<0.001	−2.553	−0.852
NIFTP	EFVPTC	−2.05 *	0.311	<0.001	−2.858	−1.479
	LT	−1.017 *	0.326	0.011	−1.864	−0.170
EFVPTC	LT	1.035 *	0.321	0.008	0.202	1.867

* The mean difference is significant at *p* < 0.05 level (F(3162) = 29.395, *p* < 0.001 one-way ANOVA).

**Table 4:** Comparison of nuclear Gal-3 expression among Benign, NIFTP, EFVPTC and Lymphocytic thyroiditis (LT).

(I) Groups	(J) Groups	Mean Difference (I–J)	Std. Error	*p* Value	95% CI	
					Lower Bound	Upper Bound
Benign	NIFTP	0.433	0.224	0.219	−0.149	1.014
	EFVPTC	−0.422	0.218	0.215	−0.987	0.142
	LT	0.183	0.229	0.855	−0.411	0.776
NIFTP	EFVPTC	−0.833 *	0.220	0.001	−1.405	−0.262
	LT	−0.228	0.231	0.758	−0.828	0.372
EFVPTC	LT	0.605 *	0.224	0.038	0.024	1.186

* The mean difference is significant at *p* < 0.05 level (F(3162) = 5.166, *p* = 0.002 one-way ANOVA).

**Table 5:** The clinical utility of biomarkers was assessed using sensitivity, specificity, positive predictive value (PPV), negative predictive value (NPV) and area under the curve (AUC) between benign or NIFTP vs. invasive EFVPTC group.

Gal-3 Staining	Sensitivity (%)	Specificity (%)	PPV (%)	NPV (%)	AUC		
					Area	*p* Value	95% CI
Benign vs. Invasive EFVPTC							
Cytoplasmic Gal-3	80.0	81.0	81.8	79.1	0.90	<0.001	0.83–0.97
Nuclear Gal-3	48.9	71.4	64.7	56.6	0.58	0.194	0.46–0.70
NIFTP vs. Invasive EFVPTC							
Cytoplasmic Gal-3	75.6	80.5	81.0	75.0	0.83	<0.001	0.75–0.92
Nuclear Gal-3	46.7	90.2	84.0	60.7	0.66	0.012	0.54–0.77

## Supplementary Materials

The following are available online at https://www.mdpi.com/article/10.3390/cancers13122988/s1, Figure S1: Photomicrographs of IHC scoring of Gal-3 expression in thyroid tissue sections. Figure S2: ROC curve analysis showed each cut-off value of IHC scores. Table S1: The cut-off values for immunopositivity of cytoplasmic Gal-3 expression were compared to Sensitivity and Specificity via ROC analysis.
