# Supplementary material for: Diagnostic Value of Galectin-3 in Distinguishing Invasive Encapsulated Carcinoma from Noninvasive Follicular Thyroid Neoplasms with Papillary-Like Nuclear Features (NIFTP)
Source: Cancers (Basel). 2021 Jun 15;13(12):2988. doi: 10.3390/cancers13122988 (PMC8232163; doi:10.3390/cancers13122988)
Supplement: Supplementary file 1 [file cancers-13-02988-s001.zip › cancers-1221346-supplementary.pdf]

Supplementary Figures and Table

Figure S1. Photomicrographs of IHC scoring of Gal-3 expression in thyroid tissue sections. *A*. Negative to weak cytoplasmic Gal-3 expression in thyroid nodules tissue section. (Cytoplasmic Gal-3 scoring as 0.7, Nuclear Gal-3 scoring as 0). *B.* Weak cytoplasmic Gal-3 immunostaining. (Cytoplasmic Gal-3 scoring as 1.3, Nuclear Gal-3 scoring as 0.7). *C*. Weak to mild cytoplasmic Gal-3 expression. (Cytoplasmic Gal-3 scoring as 2.6, Nuclear Gal-3 scoring as 0.3). *D*. Moderate cytoplasmic Gal-3 expression. (Cytoplasmic Gal-3 scoring as 3.3, Nuclear Gal-3 scoring as 0.7). *E*. Moderate to strong cytoplasmic Gal-3 immunostaining. (Cytoplasmic Gal-3 scoring as 4.6, Nuclear Gal-3 scoring as 2.6). *F.* Strong cytoplasmic Gal-3 immunostaining. (Cytoplasmic Gal-3 scoring as 5.6, Nuclear Gal-3 scoring as 2.0). *G.* Strong cytoplasmic Gal-3 immunostaining. (Cytoplasmic Gal-3 scoring as 6.3, Nuclear Gal-3 scoring as 4.2). *H.* Strong cytoplasmic Gal-3 immunostaining. (Cytoplasmic Gal-3 scoring as 6.6, Nuclear Gal-3 scoring as 0.3). All photomicrographs were shown at original magnification x200.

Figure S2. ROC curve analysis showed each cut-off value of IHC scores. Based upon ROC curve analysis to obtain a sensitivity 0.80 and specificity 0.81, a cut-off ≥ 3.25 was chosen as a positive criterion for cytoplasmic Gal-3 expression (see Supplementary File Table S1).

Figure S1.

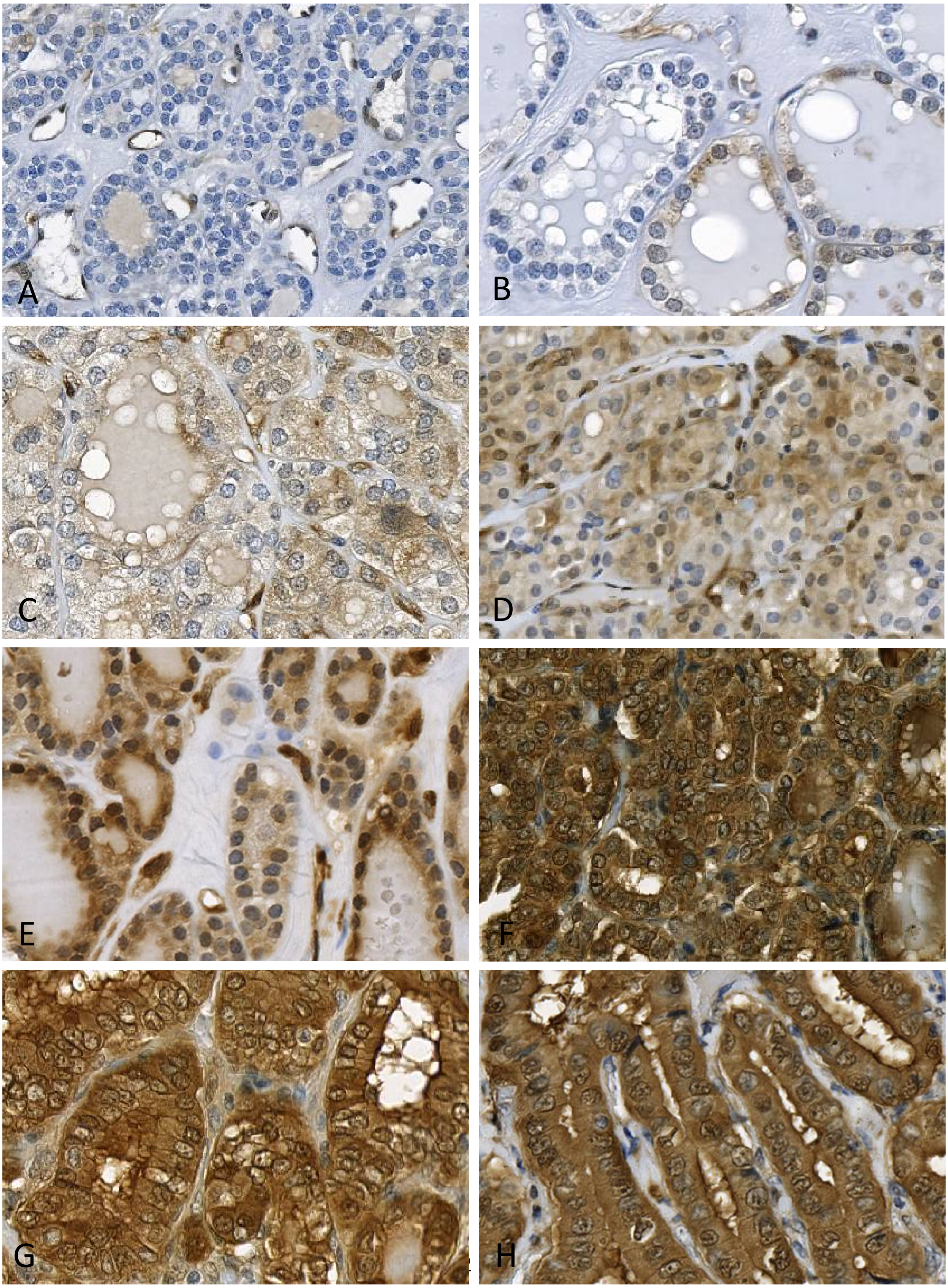

Figure S2.

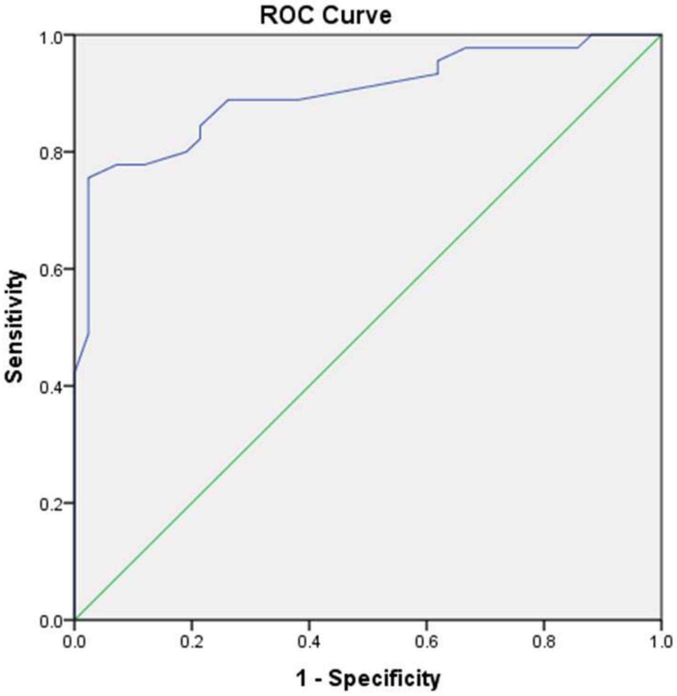

Table S1. The cut-off values for immunopositivity of cytoplasmic Gal-3 expression were compared to Sensitivity and Specificity via ROC analysis.

Positive if Greater Than or Equal To[a]	Sensitivity	Specificity
-1.0000	1.000	0.000
0.1667	1.000	0.095
0.5000	1.000	0.119
0.8333	0.978	0.143
1.0833	0.978	0.214
1.2500	0.978	0.238
1.4167	0.978	0.310
1.5833	0.978	0.333
1.7083	0.956	0.381
1.8750	0.933	0.381
2.1667	0.911	0.500
2.4167	0.889	0.619
2.5833	0.889	0.643
2.8333	0.889	0.738
3.0417	0.844	0.786
3.1250	0.822	0.786
3.2500	0.800	0.810
3.4167	0.778	0.881
3.5833	0.778	0.929
3.7083	0.756	0.976
3.7917	0.733	0.976
3.9167	0.711	0.976
4.0833	0.689	0.976
4.2500	0.667	0.976
4.4167	0.644	0.976
4.5833	0.622	0.976
4.7500	0.600	0.976
4.9167	0.578	0.976
5.0833	0.511	0.976
5.2500	0.489	0.976
5.4167	0.422	1.000
5.5833	0.400	1.000
5.7500	0.356	1.000
5.9167	0.289	1.000
6.0833	0.222	1.000
6.2500	0.178	1.000
6.5000	0.111	1.000
6.8333	0.067	1.000
8.0000	0.000	1.000

a. Cytoplasmic Gal-3 expression has at least one tie between the positive actual state group and the negative actual state group.

b. The smallest cutoff value is the minimum observed test value minus 1, and the largest cutoff value is the maximum observed test value plus 1. All the other cutoff values are the averages of two consecutive ordered observed test values. The optimum cutoff value 3.25 with a sensitivity 0.80 and specificity 0.81 was chosen for statistical analysis.
